# In Vivo Time‐Resolved Fluorescence Detection of Liver Cancer Supported by Machine Learning

**DOI:** 10.1002/lsm.23861

**Published:** 2024-11-17

**Authors:** Elena V. Potapova, Valery V. Shupletsov, Viktor V. Dremin, Evgenii A. Zherebtsov, Andrian V. Mamoshin, Andrey V. Dunaev

**Affiliations:** ^1^ Research & Development Center of Biomedical Photonics Orel State University Orel Russia; ^2^ College of Engineering and Physical Sciences Aston University Birmingham UK; ^3^ Optoelectronics and Measurement Techniques Unit University of Oulu Oulu Finland; ^4^ Orel Regional Clinical Hospital Orel Russia

**Keywords:** liver cancer, machine learning, optical biopsy, percutaneous needle biopsy, time‐resolved fluorescence

## Abstract

**Objectives:**

One of the widely used optical biopsy methods for monitoring cellular and tissue metabolism is time‐resolved fluorescence. The use of this method in optical liver biopsy has a high potential for studying the shift in energy‐type production from oxidative phosphorylation to glycolysis and changes in the antioxidant defense of malignant cells. On the other hand, machine learning methods have proven to be an excellent solution to classification problems in medical practice, including biomedical optics. We aim to combine time‐resolved fluorescence measurements and machine learning to automate the division of liver parenchyma and tumors (primary malignant, metastases and benign tumors) into classes.

**Materials and Methods:**

An optical biopsy was performed using a developed setup with a fine‐needle optical probe in clinical conditions under ultrasound control. Fluorescence decays were recorded in a conditionally healthy liver and lesions during percutaneous needle biopsy. The labeled data set was created on the basis of the recorded fluorescence results and the histopathological classification of the biopsies obtained. Several machine learning methods were trained using different separation strategies of the training test set, and their respective accuracy was compared.

**Results:**

Our results show that each of the tumor types had its own characteristic metabolic shifts recorded by the time‐resolved fluorescence spectroscopy. The application of machine learning demonstrates a reliable separation of the liver and all tumor types into cancer and noncancer classes with sensitivity, specificity and corresponding accuracy greater than 0.91, 0.79 and 0.90, using the random forest method. We also show that our method is capable of giving a preliminary diagnosis of the type of liver tumor (primary malignant, metastases and benign tumors) with a sensitivity, specificity and accuracy of at least 0.80, 0.95 and 0.90.

**Conclusions:**

These promising results highlight its potential as a key tool in the future development of diagnostic and therapeutic strategies for liver cancers. Lasers Surg. Med. 00:00–00, 2024. 2024 Wiley Periodicals LLC.

## 1 Introduction

Liver cancer is one of the most fatal cancers, and the liver is a common site for metastasis of extrahepatic tumors [1]. Differentiation of primary liver cancer from metastases is important for the selection of treatment strategies such as hepatectomy, liver transplantation, systemic treatment, as well as alternative treatment methods, including cryoablation, radiofrequency ablation, laser ablation, and so forth [2, 3, 4]. When examining patients with chronic liver disease and suspicion of liver cancer, preference is given to visualization methods including ultrasound (US), computed tomography (CT), magnetic resonance imaging (MRI), positron emission tomography (PET) and angiography [5, 6]. At the same time, the diagnostic effectiveness of these methods depends on the size of the tumor, its morphological characteristics, as well as on the diseases and conditions of the liver [7]. Since the success of liver cancer treatment depends on the early diagnosis of this disease, in most cases, patients are referred for a biopsy [8]. Percutaneous needle biopsy (PNB) of the liver is usually considered a minimally invasive and safe procedure [9]. PNB provides diagnostic information on oncology disorders (the presence of invasion, histological type of tumor, etc.), which is of primary importance in determining the treatment strategy in each clinical case and plays a critical role in the transition to personalized medicine. One of the problems in the PNB is the high probability of taking samples with a nondiagnostic value for cytology or histopathology. As the information content of the sample increases, the benefits of PNB can change the current balance between the risk and benefits of this procedure [10].

Currently, there is an active search for ways to improve the diagnostic effectiveness of PNB. One technique to confirm the location of the intraprocedural lesion is to use optical detection of Indocyanine Green (ICG) fluorescence accumulated in tumors [11]. To achieve robust results, a significant amount of fluorescence contrast should be administered intravenously to the patient, which is preferably avoided. However, living cells have intrinsic endogenous fluorescence that alternates according to the metabolic state of the tissue. Our team (Research & Development Center of Biomedical Photonics, Orel State University, Orel, Russia) seeks to solve the problem of increasing the sensitivity and specificity of the diagnosis of liver tumor during PNB. Two systems have been proposed that employ spectral and lifetime fluorescent characteristics of the tissue to distinguish liver lesions from parenchyma [12, 13]. The developed optical PNB system [14], including fluorescence and diffuse reflectance spectroscopy measurements, allowed monitoring of metabolic and morphological changes in tissues [15, 16]. The use of this system during PNB allowed us to obtain high sensitivity and specificity of differentiation between normal and pathological liver tissues [12]. This approach allows one to obtain information before the tissue sample is taken, making it possible to significantly reduce the number of false‐negative biopsies. Later, we proposed an optical PNB system that includes the time‐resolved fluorescence (TRF) spectroscopy technique. We presented novel results of TRF measurements in hepatocellular carcinoma (HCC) and healthy liver tissues obtained in a murine model and in the context of limited clinical trials [13]. The proposed classification algorithm allows us to reliably distinguish HCC, healthy liver tissue and the metabolically changed liver tissues around the tumor with high sensitivity and specificity, benefiting from the on‐the‐site characterization of the tissue on the tip of the optical needle probe before the sample is taken. This study is a continuation of the introduction of needle optical biopsy into clinical practice. Here, we consider an expanded sample of patients and explore the possibilities of various advanced machine learning (ML) methods for data classification.

The use of artificial intelligence in medicine contributes to a more accurate diagnosis to satisfy the requirements for personalized treatment. ML and deep learning approaches provide an advantage due to their potential to expand the use of massive multiparametric data, including optical data [17, 18, 19] to extract meaningful information about clinical diagnosis and patient treatment decisions. A complete review of the use of artificial intelligence in the clinical diagnosis of primary liver cancer and metastases was recently published by Bakrania et al. [20]. The diagnosis of liver tumors is based on the analysis of multidimensional clinical and pathological data, as well as CT, MRI, PET and a data set of ultrasound images. One of the promising applications of ML is PNB. Lee et al. developed an ML approach to the analysis of data obtained by thyroid fine‐needle aspiration biopsy (FNAB) [21]. As initial data for the classification of benign or malignant human thyroid nodules, clusters of thyroid cells obtained by thyroid FNAB were used. Optical biopsy gives great advantages in obtaining diagnostic information from biological tissue directly during minimally invasive surgery. Previously, we demonstrated that various ML algorithms, together with in vivo fluorescence and diffuse reflectance measurements, provide high diagnostic efficiency for binary differentiation between cancerous and healthy tissues [12, 13, 22]. However, the use of TRF, which has a high sensitivity to metabolic shifts in biological tissues [23, 24, 25, 26, 27], could possibly be extended beyond binary classification. The shift in the energy‐type production from oxidative phosphorylation to glycolysis in malignant cells, as well as changes in their antioxidant defense mechanisms, can be effectively detected by analyzing the fluorescence lifetime profile of NADH and NADPH components [26]. We suggest that the reprogrammed metabolism of various types of liver tumors can be registered with a TRF and this may become the basis for the in vivo differentiation of various types of liver cancer.

## 2 Methods and Materials

### 2.1 Time‐Resolved Fluorescence Optical Biopsy System

**Figure 1:**
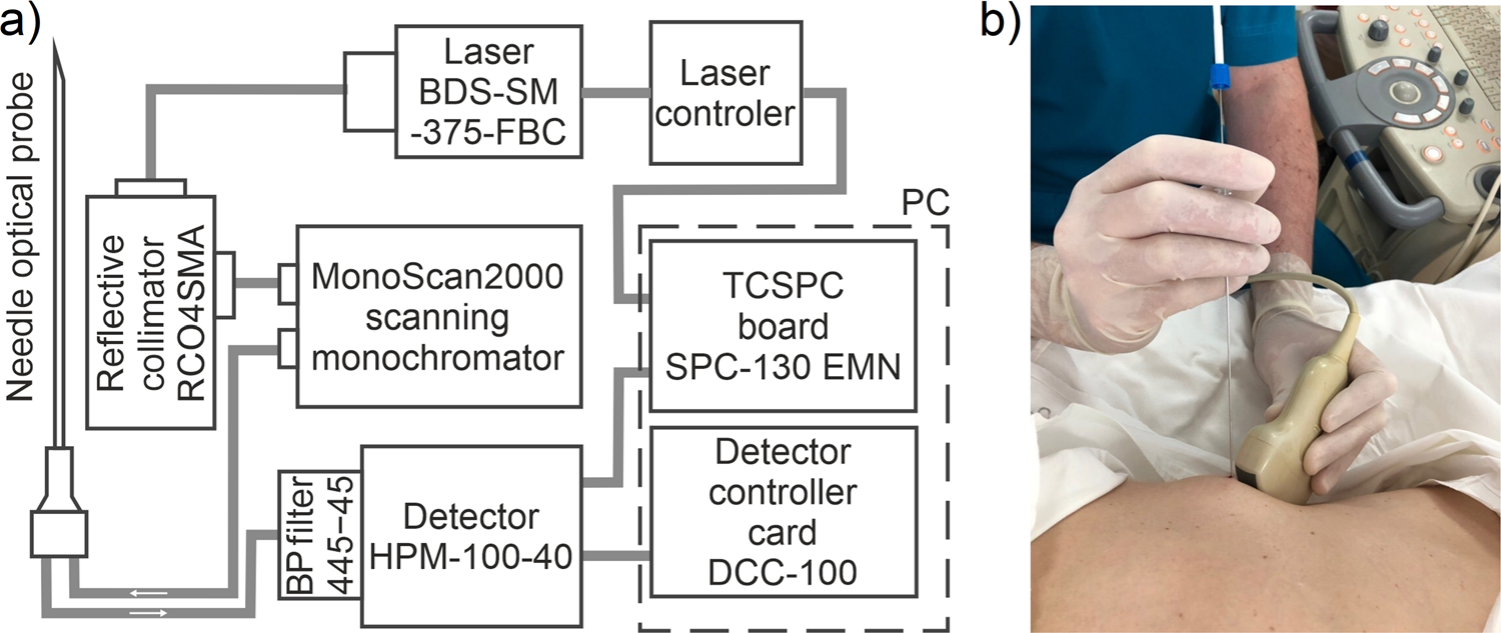
TRF optical biopsy system: (a) structural scheme of the system; (b) principle of TRF measurements during the standard PNB procedure of the liver.

In this work, we used the TRF channel of a multimodal setup and an original needle optical probe described earlier [12, 13]. The structural scheme of the TRF system and the measurement procedure are shown in Figure 1. The laser source BDS‐SM‐375‐FBC‐101 (B&H, Germany) was used for the fluorescence excitation at a wavelength of 375 nm. At this excitation, fluorescence is dominated by NAD(P)H emission. Hybrid photodetector HPM‐100‐40 (B&H, Germany) with a spectral sensitivity range of 250–720 nm and quantum efficiency of 45% (for 500 nm) was used for the photon counting. Band‐pass filter MF445‐45 (ThorLabs, USA) was used for the selection of the fluorescence emission for the recording channel. The optical power of UV radiation after the optical fiber was 0.2 mW. The exposure time for measuring one spectrum was 1 s. To validate the data obtained using the TRF optical biopsy system and the original needle optical probe, we recorded the fluorescence decay curves from fresh solutions of NADH, diluted in concentrations matching their possible concentrations in a living cell [13].

The fluorescence decay curve recorded at all points was satisfactorily described using a nonlinear least squares fitting algorithm to a two‐exponential decay model: *I(t)=Inoise* + *I*_0_ [*α*_1_ exp (−*t*/*τ*_1_) + *α*_2_ exp (−*t*/*τ*_2_)], where *I(t)* is signal intensity at time after the excitation light has ceased, counts; *τ*_1_ and *τ*_2_ are the fluorophore lifetimes, ps (*τ*_1_ is the short lifetime component and *τ*_2_ is the long lifetime component); *α*_1_ and *α*_2_ are the relative contributions of the lifetime components, % (i.e. *α*_1_ + *α*_2_ = 100%), and *I*_*noise*_ accounts for constant background signal, counts. A good fit is characterized by a *χ* ^2^ value close to 1 and residuals showing no noticeable systematic variations.

### 2.2 Clinical Study

The clinical study was conducted at the Department of Interventional Radiology of Orel Regional Clinical Hospital (Orel, Russia). The main stages of the protocol for optical PNB of the liver were as described by Dremin et al. [28]. The study was approved by the Ethics Committee of Orel State University and was carried out in accordance with the 2013 Declaration of Helsinki by the World Medical Association. The study included patients who had been recommended for a liver biopsy and agreed to participate. They signed an informed consent form before taking part in the study. Patients with cognitive deficits, psychiatric conditions, and various acute decompensated diseases were excluded from the study. The average age of 25 patients was 65 years (range from 51 to 85 years). The study included 9 men (36%) and 16 women (64%). The studies were conducted between 09/2022 and 01/2024. The results of conventional PNB and histopathological examination of the targeted tissue revealed 25 malignancies, of which 6 were classified as primary liver cancer – HCC, 6 as benign tumor (BT), and 13 as metastases (MTS). In this study of the informative material from biopsy punctures, it was sufficient in all 25 cases, and we accepted the results of the histopathological examination as 100% effective. The surgeon identified the liver lesion by ultrasound examination. The optical probe was inserted into the biopsy needle to reach the target point, and TRF measurements were performed along the needle tract. In each patient, intact liver tissue was first measured at 1–3 points (10 spectra were recorded at each point). The biopsy needle then penetrates deeper into the affected liver tissue, where 1–3 points are also measured (10 spectra at each point). The number of points for each patient is individually selected and depends on the nature of the lesion. Then, using the same needle, a biopsy of the lesion was performed at 2–4 points, taking a column of tissue.

Furthermore, after this stage of study and the development of the classifier, 2 additional patients (a 56‐year‐old man and a 74‐year‐old woman) were examined, in whom a histopathological examination identified a liver tumor as metastases.

### 2.3 Ml Model Development

**Figure 2:**
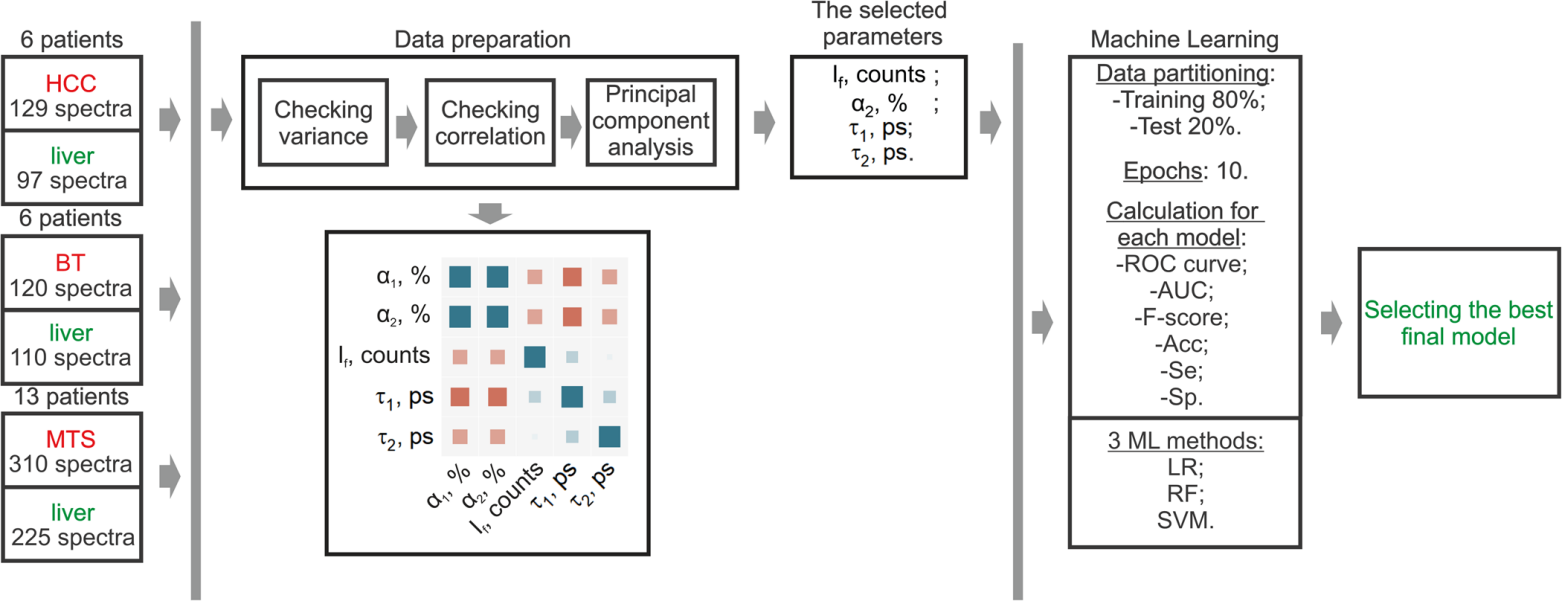
Schematic description of the training and testing process, including feature selection, different classifiers, and performance assessment.

The classification algorithm can be divided into two main components: data preparation and application of ML algorithms (Figure 2). The data preparation for all the ML methods used was the same. The first step reduces dimensionality and excludes features that have a negligible impact on further learning. Thus, the algorithm starts by examining the variance of each feature in the data set. Features with low variance ( < 1) are excluded from further analysis. The algorithm then evaluates the Pearson correlations between all pairs of features. Features with significant correlation ( > 0.7) are excluded, as they can introduce redundancy and potentially distort the learning process.

At this stage, one parameter was excluded–the relative contributions of the short lifetime component (*α*_1_). With the equivalent contribution of components *α*_1_ and *α*_2_ to the classification efficiency, preference was given to the *α*_2_ parameter. This is because the quantum yield of NADH increases by an order of magnitude upon binding to enzymes, and this parameter can provide more physiological information when building classifiers [29, 30]. Also, in our previous work, we demonstrated the highest diagnostic significance of this parameter for differentiating murine liver from HCC tumors [13]. Next, principal component analysis (PCA) was applied to the remaining 4 features to potentially reduce dimensionality while preserving the maximum amount of information. This transforms the original features into a new set of uncorrelated variables called principal components. The principal components are ordered by the amount of variance they explain, allowing the most informative components to be selected. All 4 parameters explain the total variance equally (∼25%). Therefore, after completion of the data preparation part, 4 parameters were selected for ML: fluorescence intensity (*I*_*f*_), the relative contributions of the long lifetime component (*α*_2_), short fluorescence lifetime (*τ*_1_), long fluorescence lifetime (*τ*_2_). The selected parameters were the final input to the ML model.

To train and evaluate our models, we used data partitioning, with 80% data for training and 20% for testing. This separation allowed us to train the models on a significant amount of data and independently evaluate their performance. During the training phase, the algorithm spent 10 epochs randomly mixing the data into training and test sets. This iterative process allowed the models to learn from the data and improve their predictive capabilities. It should be noted here that spectra from one patient can appear in both the test and training sets. But in our case, it should not lead to data leakage because spectra within one sample are different from each other. As we mentioned above, this is due to the measurement of parameters along the biopsy needle tract.

The developed model utilizes random forest (RF), support vector machine (SVM), and logistic regression (LR) methods, which are the most commonly used to solve similar problems.

In our model, the RF ML method uses bootstrap aggregation, also known as bagging, to build an ensemble of decision trees. Using the bootstrap method, subsets of the original training sample are randomly selected (100 estimators) with repetitions. For each subset of training data, a decision tree is constructed, which is trained independently and uses only some randomly selected features from the total feature set. In an RF, a decision is made by aggregating the average of the decisions of all trees. Thus, the classification result is obtained by combining the predictions of all trees.

The second ML method we used was the support vector classifier (SVC), which is a variation of SVM and allowed us to deal with nonlinear data. It transforms the original data space into a new, higher‐dimensional one where the data become linearly separable. SVC then applies the SVM method to the new spatial data. In the algorithm developed, the first step is data preparation, which includes feature scaling and splitting the data into training and test samples. Then, an SVC model with a polynomial kernel is created which is trained on the training sample. During training, SVC finds the optimal hyperplane to maximize the class gap.

As the third ML method, we used the LR method, which is based on a logistic function to predict the probability that an object belongs to a certain class. This method is based on linear regression, but uses a sigmoid function, also known as a logistic function, to obtain class probabilities. In our approach, the model was trained on a training sample where the coefficients of the linear combination of features were adjusted using the maximum likelihood method. Once trained, the model was used to classify new data by predicting the probability of belonging to each class.

In this paper, we used binary (liver/tumor) and multi‐class (BT/MTS/HCC) classification models. For binary classification, the two classes were compared with each other, namely BT (*n* = 120)/liver (*n* = 110), HCC (*n* = 129)/liver (*n* = 97) and MTS (*n* = 310)/liver (*n* = 225), calculating the evaluation metrics for each pair. For multi‐class classification, 3 tumor types were compared with each other, namely BT (*n* = 120)/HCC (*n* = 129)/MTS (*n* = 310), calculating evaluation metrics for each class.

To assess the performance of each model, we employed several evaluation metrics (see Figure 2). The ROC‐curve illustrates the trade‐off between the true positive rate and the false positive rate at various classification thresholds. This provides information on the discriminatory power and performance of the model. The *AUC* metric quantifies the overall performance of the model by measuring the area under the ROC‐curve. A higher AUC value indicates better discriminative ability and model performance. The *F‐score* combines precision and recall to measure the accuracy of the model in binary classification tasks. This provides a balanced assessment of the model's performance. *Accuracy* represents the ratio of correctly classified instances to the total number of instances. It provides a general overview of the model's predictive accuracy. *Sensitivity*, also known as the recall or true positive rate, measures the model's ability to classify positive instances correctly. *Specificity* quantifies the model's ability to classify negative instances correctly.

## 3 Results and Discussion

**Figure 3:**
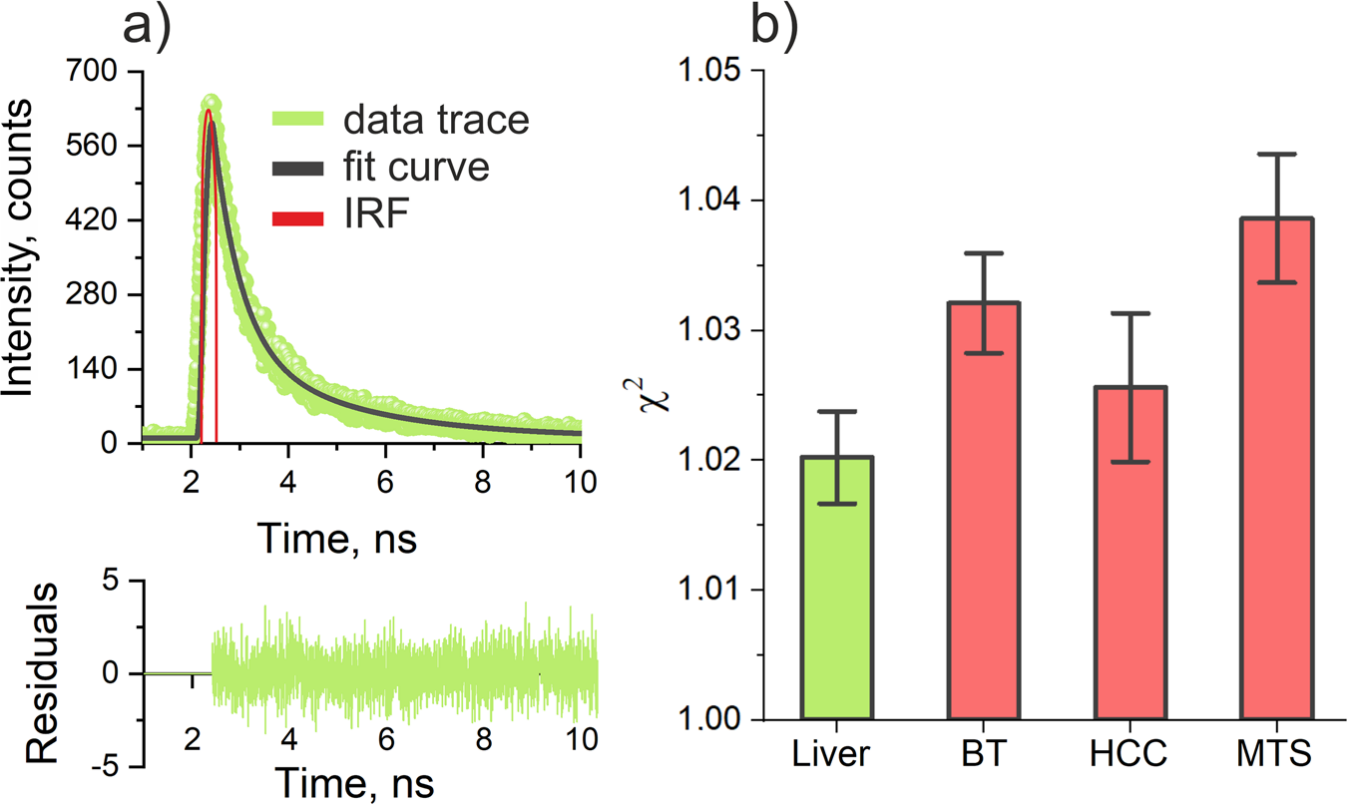
Relevant traces of fluorescence decay recorded by the TRF setup through the needle optical probe in the liver: (a) a biexponential decay model adequately describing the fluorescence decay with instrumental responding function (IRF); (b) *χ* ^2^‐values obtained in the fitting.

**Figure 4:**
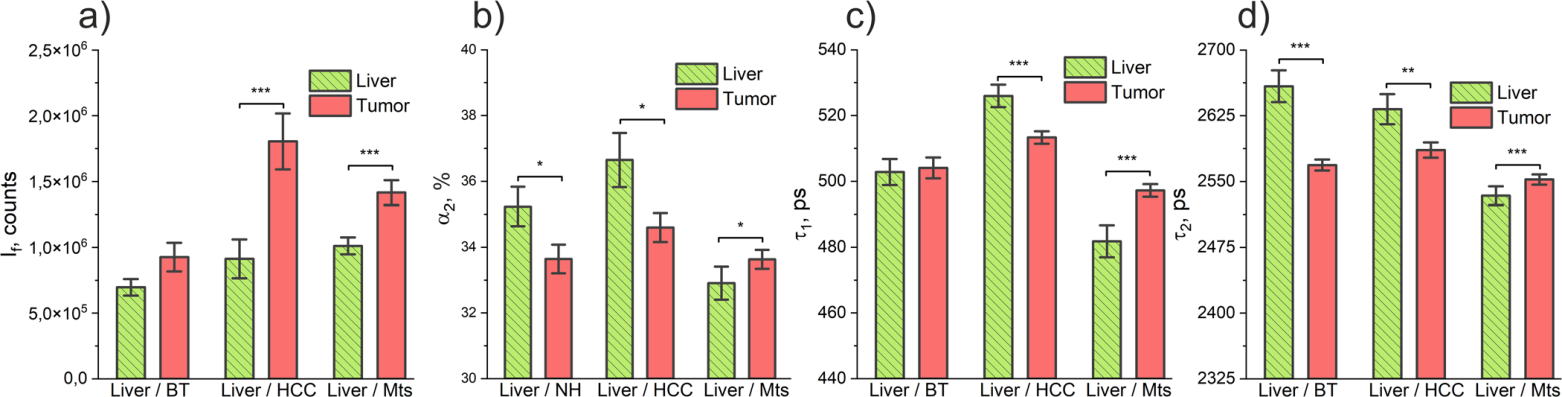
Parameters evaluated by the fluorescence lifetime measurements through the needle optical probe: (a) total fluorescence intensity *I*_*f*_; (b) the amplitude of the long decay component, *α*_2_; (c) short fluorescence lifetime, *τ*_1_; (d) long fluorescence lifetime, *τ*_2_. * – *p* < 0.05; ** – *p* < 0.01; *** – *p* < 0.001. A Mann–Whitney *U*‐test was used to identify differences between the groups.

Figure 3a shows representative traces of fluorescence decay curves registered in the liver parenchyma, as well as the applied two‐exponential fitting decay model adequately describing the process. *χ* ^2^ values obtained from the fitting of the decay curves are shown in Figure 3b. Here, and further, the graphs and text provide information on the average and standard error (SE) of the values analyzed. The results of TRF obtained in this clinical study (see Figure 4) are largely consistent with the preclinical research on liver tumors using the TRF method we previously described [13].

The fluorescence intensity *I*_*f*_ measured in the malignant tumor was higher than in the liver tissues (Figure 4a): liver/HCC (0.91·10^6^ ± 0.09·10^6^ counts and 1.80·10^6^ ± 0.01·10^6^ counts correspondingly, *p* < 0.001); liver/MTS (1.01·10^6^ ± 0.04·10^6^ counts and 1.42·10^6^ ± 0.63·10^6^ counts correspondingly, *p* < 0.001). This can be explained by a significant shift in tumor metabolism, that is, the general accumulation of NAD(P)H. If we talk about primary liver cancer (HCC), the hypothesis of a shift in cellular metabolism toward glycolysis and/or lower mitochondrial respiration is confirmed by the decrease in protein‐bound NAD(P)H lifetime *τ*_2_ in HCC, compared to the liver parenchyma (2585 ± 6 ps and 2632 ± 10 ps, respectively, *p* < 0.01, Figure 4d) and the decrease the relative contributions of the long lifetime component α_2_ (HCC [34.59% ± 0.29%], liver parenchyma [36.65% ± 0.55%], *p* < 0.05, Figure 4b). In a typical case, if other metabolic pathways do not have a significant effect, a change in the ratio of the relative contributions of free and bound forms is observed due to a change in the balance between glycolysis and oxidative phosphorylation [31]. These data confirm previously obtained results about metabolic remodeling in HCC involving a significant increase in glycolysis over mitochondrial oxidative phosphorylation [26, 32, 33].

The TRF results obtained for the group of patients with MTS showed an increase in the relative contributions of the long lifetime component *α*_2_ in MTS (33.63% ± 0.19%) relative to the liver parenchyma (32.91 ± 0.34%), *p* < 0.05, Figure 4b. At the same time, we observed an increased lifetime in MTS of the long lifetime *τ*_2_ (MTS vs. liver: 2552 ± 4 ps vs. 2533 ± 7 ps, *p* < 0.001, Figure 4d). This allows us to assume that metabolism in MTS does not have a predominant glycolytic pathway and may be due to other ways of metabolic rearrangement. Metabolic stress inevitably causes ROS‐mediated oxidative stress, even under hypoxic conditions, which makes NAD(P)H homeostasis critical for cell survival [34]. Protein‐bound NAD(P)H has a longer lifetime and may explain the results observed. This hypothesis requires additional verification. Furthermore, the studied group of patients with MTS had different primary tumors, as well as different differentiation of the tumors themselves in histological samples. This also requires an additional data set and may form the basis for the development of MTS differentiation technology in the future.

In the group of patients with benign liver tumor (BT), the difference in fluorescence intensity and short lifetime *τ*_1_ did not reach statistical significance (Figure 4a,c). According to 18‐Fluoro‐2‐deoxyglucose‐positron emission tomography/computed tomography, in most benign tumors and nontumorous lesions, there are no changes in the cellular metabolism of glucose [35]. A decrease in long lifetime *τ*_2_ in BT 2569 ± 10 ps compared to 2659 ± 10 ps in the liver (*p* < 0.001, Figure 4d) and the relative contributions of the long lifetime component *α*_2_ in BT (33.64% ± 0.29%) regarding liver tissue (35.26% ± 0.39%) (*p* < 0.001, Figure 4b) may indirectly indicate that these types of tumor also have metabolic rearrangements, but this requires additional research.

We tested the classification efficiency under the linear combination of features using linear discriminant analysis (LDA). The results are presented in Table 1. The parameters *α*_2_ and *τ*_2_ were chosen as having the highest classification ability according to (Figure 4b and Figure 4d). The table summarizes the results for sensitivity, specificity, accuracy, and F‐score. The results are presented as a mean value ± standard deviation. The calculated accuracies were small for reliable clinical use, which confirms the need for more complex ML methods.

**Table 1:** The results of the LDA classification.

BT/Liver	HCC/Liver	MTS/Liver
Se: 0.84 ± 0.10	Se: 0.76 ± 0.08	Se: 0.76 ± 0.04
Sp: 0.76 ± 0.14	Sp: 0.71 ± 0.12	Sp: 0.61 ± 0.05
Acc: 0.79 ± 0.06	Acc: 0.74 ± 0.05	Acc: 0.70 ± 0.03
F‐score: 0.80 ± 0.06	F‐score: 0.76 ± 0.04	F‐score: 0.74 ± 0.03
AUC: 0.82 ± 0.07	AUC: 0.73 ± 0.08	AUC: 0.72 ± 0.03

Abbreviations: Acc, accuracy; F‐score, measure of predictive performance; AUC, area under the curve; Se, sensitivity; Sp, specificity.

In the first stage of the ML application, we developed a classifier to differentiate the liver parenchyma from various types of tumors. For this binary classification, we compared a set of spectra of patients with one histologically confirmed diagnosis and a set of liver spectra of the same patients. Here, it is worth noting that the accuracy values for the training sample for all classification pairs were high values, at least 0.98. Table 2 summarizes the results that we achieved from 10 statistical experiments where the data used for training and testing were randomly shuffled each time.

**Table 2:** The results of the differentiation of liver parenchyma and different types of tumor.

	BT/Liver	HCC/Liver	MTS/Liver
SVM	Se: 0.40 ± 0.22	Se: 0.75 ± 0.08	Se: 0.80 ± 0.07
	Sp: 0.72 ± 0.28	Sp: 0.61 ± 0.14	Sp: 0.31 ± 0.07
	Acc: 0.53 ± 0.07	Acc: 0.68 ± 0.09	Acc: 0.60 ± 0.03
	F‐score: 0.45 ± 0.11	F‐score: 0.72 ± 0.08	F‐score: 0.71 ± 0.03
	AUC:0.47 ± 0.13	AUC: 0.73 ± 0.09	AUC: 0.67 ± 0.05
LR	Se: 0.60 ± 0.23	Se: 0.65 ± 0.09	Se: 0.69 ± 0.10
	Sp: 0.58 ± 0.18	Sp: 0.56 ± 0.14	Sp: 0.47 ± 0.07
	Acc: 0.57 ± 0.07	Acc: 0.60 ± 0.08	Acc: 0.60 ± 0.04
	F‐score: 0.57 ± 0.14	F‐score: 0.64 ± 0.08	F‐score: 0.67 ± 0.04
	AUC: 0.64 ± 0.11	AUC: 0.68 ± 0.12	AUC: 0.64 ± 0.05
RF	Se: 0.97 ± 0.04	Se: 0.97 ± 0.03	Se: 0.94 ± 0.04
	Sp: 0.87 ± 0.05	Sp: 0.81 ± 0.06	Sp: 0.84 ± 0.05
	Acc: 0.92 ± 0.03	Acc: 0.90 ± 0.03	Acc: 0.90 ± 0.03
	F‐score: 0.93 ± 0.03	F‐score: 0.91 ± 0.03	F‐score: 0.92 ± 0.02
	AUC: 0.99 ± 0.02	AUC: 0.98 ± 0.01	AUC: 0.98 ± 0.01

**Figure 5:**
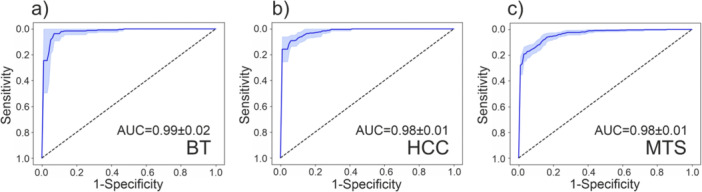
ROC‐curves for assessing the effectiveness of RF‐based classifiers: (a) BT/liver; (b) HCC/liver; (c) MTS/liver.

From all considered values of accuracy, the RF algorithm for the TRF data provides the best performance for the differentiation of the liver parenchyma and all types of tumors (0.90–0.92). At the same time, the best values of sensitivity and specificity of the developed classifier are observed to separate the classes of liver parenchyma and benign tumor–BT (0.97 ± 0.04 and 0.87 ± 0.05), as well as liver parenchyma and primary liver tumor–HCC (0.97 ± 0.03 and 0.81 ± 0.06). This is also confirmed by the high values of the F‐score metric (0.91 ± 0.03). The lower values of the diagnostic efficiency metrics of the developed classifier for groups of liver parenchyma and MTS can be explained by a large number of different types of MTS in this group of patients. The ROC‐curves to assess the effectiveness of the classifier are shown in Figure 5.

We also performed experiments with an RF algorithm to classify tumor types in vivo on the basis of TRF measurement. The parameters of the obtained model are summarized in Table 3. In this classification algorithm, each tumor type is compared with two other types. Accordingly, the presented accuracy values show the algorithm's ability to distinguish a specific type of tumor from two other types. The task of classifying tumors into different groups was more difficult, but diagnostic accuracy metrics reached high values for all types of cancer: 0.90–0.95. The detection of HCC with the most pronounced and unequivocal shifts in tissue metabolism registered by the TRF method has the highest sensitivity (0.91 ± 0.08) and specificity (0.96 ± 0.02) in the classification of tumor types.

**Table 3:** The results of the classification of different types of tumor.

	BT	HCC	MTS
RF	Se: 0.84 ± 0.07	Se: 0.91 ± 0.08	Se: 0.92 ± 0.03
	Sp: 0.97 ± 0.01	Sp: 0.96 ± 0.02	Sp: 0.88 ± 0.05
	Acc: 0.95 ± 0.01	Acc: 0.95 ± 0.03	Acc: 0.90 ± 0.03
	F: 0.88 ± 0.03	F: 0.89 ± 0.04	F: 0.91 ± 0.04
	AUC: 0.89 ± 0.06	AUC: 0.94 ± 0.08	AUC: 0.90 ± 0.03

After the algorithm was developed, two patients with suspected liver tumors were admitted to the clinic. They also underwent an optical biopsy procedure with registration and analysis of TRF signals. The classifier we developed showed that for the patients, the probability of an MTS diagnosis was 80%. This diagnosis was later confirmed by the results of the histopathological examination, which showed that the diagnoses were indeed accurate.

Despite the achievements in improving multiphase CT or MRI methods, the diagnosis of liver cancer cannot be confirmed by imaging methods in some patients (e.g., without cirrhosis of the liver), despite the enhanced brightness in radiology tests and liver biopsy is required in these cases. Over the past decade, breakthroughs in artificial intelligence have inspired researchers to develop algorithms for the diagnosis of liver cancer. The technology of optical biopsy data processing with ML, developed by us, is as accurate in diagnostics as the proposed algorithms for processing PET, MRI, CT, US imaging, and histology data [20]. In this paper, we have shown that our proposed TRF‐based classification of liver cancer supported by ML technology not only allows a solution to the important problem of choosing the biopsy sampling site when performing PNB but also can immediately give a preliminary diagnosis of the benign or malignant nature of the tumor, as well as its origin (primary tumor or metastases). It is also worth noting that there are other optical biopsy methods that have a high diagnostic potential. To date, experience has accumulated in creating devices for fine needle biopsy based on Raman spectroscopy [36], OCT [37, 38, 39], fluorescence spectroscopy [40, 41], diffuse reflectance spectroscopy [42, 43]. But at the moment, these methods have not been used to solve the problem of classifying different types of tumors.

## 4 Conclusion

This study provided a comprehensive analysis of fluorescence decay curves in liver tumors and liver parenchyma, with particular emphasis on the results derived from the ML classification. The two‐exponential fitting decay model was found to be a robust descriptor of the fluorescence decay process in these tissues. A significant observation was the elevated fluorescence intensity, denoted by *I*_*f*_, in malignant tumors compared to liver tissues, pointing to a pronounced shift in tumor metabolism, especially the accumulation of NADH and NADPH.

The ML classifiers further enriched our findings. SVM, LR, and RF models, among others, showcased varying degrees of sensitivity and specificity in distinguishing between different tumor types and liver tissues. These classifiers highlighted the potential of ML to enhance the diagnostic precision of fluorescence‐based techniques. For example, the RF model exhibited high sensitivity and specificity among different tumor types, underscoring its potential as a reliable diagnostic tool.

Although primary liver tumors (HCC) indicated a metabolic shift toward glycolysis, the TRF results for MTS patients presented a more complex picture, suggesting various metabolic pathways in MTS compared to primary tumors. In patients with benign liver neoplasms (BT), the ML classifiers revealed subtle differences in fluorescence lifetime parameters, reinforcing the notion that benign tumors and nontumorous lesions exhibit minimal changes in glucose cellular metabolism.

Of course, our approach to data analysis has limitations similar to those of any artificial intelligence application for medical diagnosis and treatment. The main reasons for poor model accuracy and inaccurate predictions of results are either insufficient or excessive training of the data. The small sample size in this study may have affected the accuracy parameters obtained, although we attempted to address this issue. The group of patients is not a large enough sample, but the data obtained during the optical biopsy are unique, and the method of expanding the sample by registering signals during the moving of the biopsy needle at several points of the liver parenchyma and tumor is acceptable for such studies. We plan to continue our research in several clinics to conduct a multicenter clinical trial and minimize possible systematic errors in the data set.

The promising results of the ML classification highlight its potential as a key tool in the future development of diagnostic and therapeutic strategies for liver tumors. Our current results show that the integration of TRF into biopsy needles may be useful for characterizing liver tumors in vivo and real‐time during PNB. This approach has several potential benefits including real‐time feedback: (1) to reduce the frequency of taking uninformative samples of neoplasms during puncture biopsy, leading to more accurate diagnoses and better treatment decisions for patients; (2) previously, already at the stage of biopsy, to establish a diagnosis for an earlier decision by the doctor on further treatment of patients (especially if these are metastases that were not detected earlier by other types of imaging techniques and a search for a primary tumor is necessary); (3) with further development of the method, it is possible to assess targeted areas for the presence of tumor tissue and metabolic activity after chemotherapy or the use of methods of local destruction of liver tumors.

## Conflicts of Interest

The authors declare no conflicts of interest.
